# 2-(6-Methyl-2,3,4,9-tetra­hydro-1*H*-carbazol-1-yl­idene)propane­dinitrile

**DOI:** 10.1107/S160053681104654X

**Published:** 2011-11-12

**Authors:** M. Sekar, R. Velmurugan, A. Chandramohan, P. Ramesh, M. N. Ponnuswamy

**Affiliations:** a Post Graduate and Research Department of Chemistry, Sri Ramakrishna Mission Vidyalaya College of Arts and Science, Coimbatore 641 020, India; b Centre of Advanced Study in Crystallography and Biophysics, University of Madras, Guindy Campus, Chennai 600 025, India

## Abstract

In the title compound, C_16_H_13_N_3_, the cyclo­hexene ring adopts a sofa conformation. An intra­molecular N—H⋯N hydrogen bond generates an *S*(7) ring motif. In the crystal, the mol­ecules are linked by pairs of N—H⋯N inter­actions, forming centrosymmetric dimers with an *R*_2_^2^(14) motif.

## Related literature

For the biological activity of carbazole derivatives, see: Magnus *et al.* (1992 ▶); Abraham (1975 ▶); Saxton (1983 ▶); Phillipson & Zenk (1980 ▶); Bergman & Pelcman (1990 ▶); Bonesi *et al.* (2004 ▶); Chakraborty *et al.* (1965 ▶); Kirtikar & Basu (1933 ▶); Chakraborty *et al.* (1973 ▶). For puckering parameters, see: Cremer & Pople (1975 ▶). For asymmetry parameters, see: Nardelli (1983 ▶). For hydrogen-bond motifs, see: Bernstein *et al.* (1995 ▶).
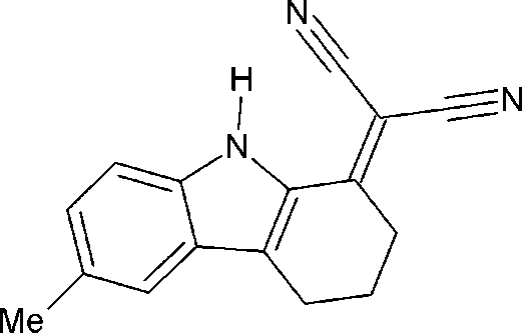


## Experimental

### 

#### Crystal data

C_16_H_13_N_3_
*M* *_r_* = 247.29
Triclinic, 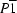

*a* = 7.6396 (9) Å
*b* = 8.4381 (8) Å
*c* = 10.8967 (13) Å
α = 88.395 (6)°
β = 71.392 (7)°
γ = 71.217 (6)°
*V* = 628.08 (12) Å^3^
*Z* = 2
Mo *K*α radiation
μ = 0.08 mm^−1^
*T* = 296 K
0.17 × 0.16 × 0.15 mm

#### Data collection

Bruker SMART APEX CCD detector diffractometer
Absorption correction: multi-scan (*SADABS*; Bruker, 1998 ▶) *T*_min_ = 0.986, *T*_max_ = 0.988
12507 measured reflections
3692 independent reflections
2815 reflections with *I* > 2σ(*I*)
*R*_int_ = 0.026

#### Refinement

*R*[*F* ^2^ > 2σ(*F* ^2^)] = 0.050
*wR*(*F* ^2^) = 0.154
*S* = 1.03
3692 reflections
177 parameters
H atoms treated by a mixture of independent and constrained refinement
Δρ_max_ = 0.32 e Å^−3^
Δρ_min_ = −0.19 e Å^−3^

### 

Data collection: *SMART* (Bruker, 1998 ▶); cell refinement: *SAINT-Plus* (Bruker, 1998 ▶); data reduction: *SAINT-Plus*; program(s) used to solve structure: *SHELXS97* (Sheldrick, 2008 ▶); program(s) used to refine structure: *SHELXL97* (Sheldrick, 2008 ▶); molecular graphics: *ORTEP-3* (Farrugia, 1997 ▶); software used to prepare material for publication: *SHELXL97* and *PLATON* (Spek, 2009 ▶).

## Supplementary Material

Crystal structure: contains datablock(s) global, I. DOI: 10.1107/S160053681104654X/bt5683sup1.cif

Structure factors: contains datablock(s) I. DOI: 10.1107/S160053681104654X/bt5683Isup2.hkl

Supplementary material file. DOI: 10.1107/S160053681104654X/bt5683Isup3.cml

Additional supplementary materials:  crystallographic information; 3D view; checkCIF report

## Figures and Tables

**Table 1:** Hydrogen-bond geometry (Å, °)

*D*—H⋯*A*	*D*—H	H⋯*A*	*D*⋯*A*	*D*—H⋯*A*
N1—H1⋯N16	0.86 (2)	2.59 (2)	3.3099 (17)	141.4 (16)
N1—H1⋯N16^i^	0.86 (2)	2.49 (2)	3.2150 (17)	142.8 (16)

Symmetry code: (i) 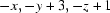.

**Fig. 1.**
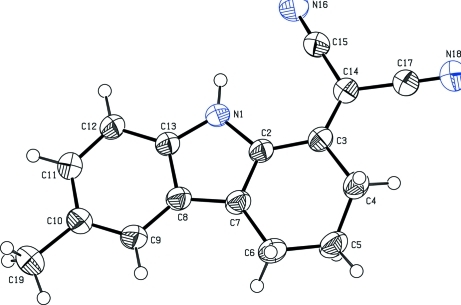
The molecular structure of the title compound, showing the atomic numbering and displacement ellipsoids drawn at the 50% probability level.

**Fig. 2.**
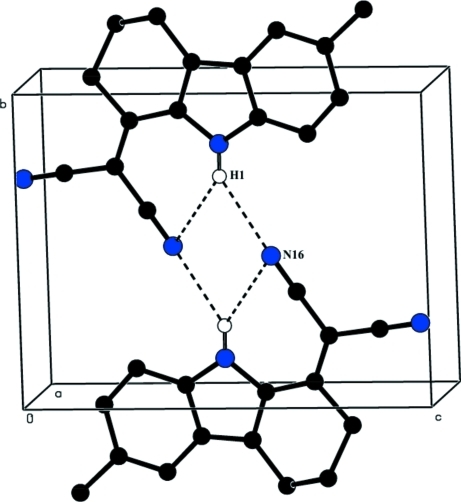
The crystal packing of the title compound. H atoms not involved in hydrogen bonding (dashed lines) have been omitted for clarity.

## supplementary crystallographic information

### Comment

Carbazole alkaloids obtained from naturally occurring sources have been the subject of extensive research, mainly because of their widespread applications in traditional medicine (Bergman & Pelcman, 1990; Bonesi *et al.*, 2004; Chakraborty *et al.*, 1965; Kirtikar & Basu, 1933). Tetrahydrocarbazole systems are present in the framework of a number of indole-type alkaloids of biological interest (Magnus *et al.*, 1992; Abraham, 1975; Saxton, 1983; Phillipson *et al.*, 1980). These types of compounds possess significant antibiotic, anti-carcinogenic, antiviral and anti-inflammatory properties (Chakraborty *et al.*, 1973). Against this background and to ascertain the molecular structure and conformation, the X-ray crystal structure determination of the title compound has been carried out.

The *ORTEP* plot of the molecule is shown in Fig. 1. The cyclohexane ring in the carbazole ring system adopts envelope conformation with the puckering parameters (Cremer & Pople, 1975) and the asymmetry parameters (Nardelli, 1983) are: q_2_=0.330 (1) Å, q_3_ = 0.262 (1) Å, φ_2_ = 168.7 (2)° and Δ_s_(C2 & C5)= 8.6 (2)°. The sum of the bond angles around N1 [359.8°] is in accordance with *sp^2^* hybridization. The bond lengths and bond angles of (C15—N16) 1.148 (2) Å, (C17—N18) 1.145 (2) Å, (C14—C15—N16) 178.8 (2)° and (C14—C17—N18) 178.6 (2)° show linear character of the cyano group, a feature observed in carbonitrile compounds.

The crystal packing reveals that symmetry-related molecules are linked by N—H···N interactions. The intramolecular N1—H1···N16 hydrogen bond generates a S(7) ring motif. The molecules at (*x*, *y*, *z*) and (2 - *x*, -1 - *y*, 1 - *z*) are linked by N1—H1···N16 hydrogen bonds into cyclic centrosymmetric *R*_2_^2^(14) dimer.

### Experimental

A mixture of 6-methyl-1-oxo-1,2,3,4-tetrahydrocarbazole (7.5 mmol), and melanonitrile (7.5 mmol), ammonium acetate (0.57 g, 8.125 mmol) and acetic acid (1.5 ml, 24.75 mmol) in 12.5 ml of toluene was stirred at 105°C for 5 h. On cooling the precipitate that formed was filtered off, washed with hexane (20 ml) and dried at 100°C to give a crude product of 6-Methyl-2-(1,2,3,4- tetrahydro-9*H*-carbazol-1-ylidene)propanedinitrile.The crystals of the title compound suitable for single XRD analysis were obtained by the slow evaporation method by using dichloroethane as solvent at room temperature.

### Refinement

The N-bound H atom was located in a difference map and refined isotropically. C-bound H atoms were positioned geometrically (C–H = 0.93–0.97 Å) and allowed to ride on their parent atoms, with *U*_iso_(H) = 1.5*U*_eq_(C) for methyl H atoms and 1.2*U*_eq_(C) for all other H atoms.

### Crystal data

**Table:**

C_16_H_13_N_3_	*Z* = 2
*M**_r_* = 247.29	*F*(000) = 260
Triclinic, *P*1	*D*_x_ = 1.308 Mg m^−^^3^
Hall symbol: -P 1	Mo *K*α radiation, λ = 0.71073 Å
*a* = 7.6396 (9) Å	Cell parameters from 1675 reflections
*b* = 8.4381 (8) Å	θ = 2.0–30.6°
*c* = 10.8967 (13) Å	µ = 0.08 mm^−^^1^
α = 88.395 (6)°	*T* = 296 K
β = 71.392 (7)°	Block, brown
γ = 71.217 (6)°	0.17 × 0.16 × 0.15 mm
*V* = 628.08 (12) Å^3^	

### Data collection

**Table:**

Bruker SMART APEX CCD detector diffractometer	3692 independent reflections
Radiation source: fine-focus sealed tube	2815 reflections with *I* > 2σ(*I*)
graphite	*R*_int_ = 0.026
ω scans	θ_max_ = 30.6°, θ_min_ = 2.0°
Absorption correction: multi-scan (*SADABS*; Bruker, 1998)	*h* = −10→10
*T*_min_ = 0.986, *T*_max_ = 0.988	*k* = −12→11
12507 measured reflections	*l* = −15→15

### Refinement

**Table:**

Refinement on *F*^2^	Primary atom site location: structure-invariant direct methods
Least-squares matrix: full	Secondary atom site location: difference Fourier map
*R*[*F*^2^ > 2σ(*F*^2^)] = 0.050	Hydrogen site location: inferred from neighbouring sites
*wR*(*F*^2^) = 0.154	H atoms treated by a mixture of independent and constrained refinement
*S* = 1.03	*w* = 1/[σ^2^(*F*_o_^2^) + (0.0895*P*)^2^ + 0.0632*P*] where *P* = (*F*_o_^2^ + 2*F*_c_^2^)/3
3692 reflections	(Δ/σ)_max_ < 0.001
177 parameters	Δρ_max_ = 0.32 e Å^−^^3^
0 restraints	Δρ_min_ = −0.19 e Å^−^^3^

### Special details

**Table:**

Geometry. All e.s.d.'s (except the e.s.d. in the dihedral angle between two l.s. planes) are estimated using the full covariance matrix. The cell e.s.d.'s are taken into account individually in the estimation of e.s.d.'s in distances, angles and torsion angles; correlations between e.s.d.'s in cell parameters are only used when they are defined by crystal symmetry. An approximate (isotropic) treatment of cell e.s.d.'s is used for estimating e.s.d.'s involving l.s. planes.
Refinement. Refinement of *F*^2^ against ALL reflections. The weighted *R*-factor *wR* and goodness of fit *S* are based on *F*^2^, conventional *R*-factors *R* are based on *F*, with *F* set to zero for negative *F*^2^. The threshold expression of *F*^2^ > σ(*F*^2^) is used only for calculating *R*-factors(gt) *etc*. and is not relevant to the choice of reflections for refinement. *R*-factors based on *F*^2^ are statistically about twice as large as those based on *F*, and *R*- factors based on ALL data will be even larger.

### Fractional atomic coordinates and isotropic or equivalent isotropic displacement parameters (Å^2^)

**Table:**

	*x*	*y*	*z*	*U*_iso_*/*U*_eq_	
N1	0.19262 (15)	1.14409 (12)	0.48492 (9)	0.0374 (2)	
H1	0.140 (3)	1.251 (3)	0.4900 (18)	0.073 (5)*	
C2	0.16168 (16)	1.03889 (13)	0.58442 (11)	0.0348 (2)	
C3	0.02788 (16)	1.08208 (13)	0.71382 (10)	0.0352 (2)	
C4	0.04245 (19)	0.94137 (16)	0.80188 (12)	0.0446 (3)	
H4A	−0.0838	0.9623	0.8687	0.054*	
H4B	0.1358	0.9420	0.8447	0.054*	
C5	0.1046 (2)	0.76799 (16)	0.73322 (13)	0.0480 (3)	
H5A	0.1234	0.6840	0.7947	0.058*	
H5B	0.0007	0.7603	0.7033	0.058*	
C6	0.29157 (19)	0.73000 (15)	0.61842 (13)	0.0452 (3)	
H6A	0.4026	0.7046	0.6494	0.054*	
H6B	0.3079	0.6323	0.5656	0.054*	
C7	0.28556 (17)	0.87609 (14)	0.53817 (11)	0.0370 (2)	
C8	0.39448 (16)	0.88053 (13)	0.40647 (11)	0.0363 (2)	
C9	0.53599 (18)	0.75647 (15)	0.30953 (12)	0.0429 (3)	
H9	0.5779	0.6452	0.3286	0.051*	
C10	0.61190 (18)	0.80110 (15)	0.18626 (12)	0.0430 (3)	
C11	0.54686 (18)	0.97060 (16)	0.15997 (12)	0.0427 (3)	
H11	0.5987	0.9992	0.0763	0.051*	
C12	0.41057 (17)	1.09577 (15)	0.25181 (11)	0.0400 (3)	
H12	0.3713	1.2069	0.2319	0.048*	
C13	0.33323 (16)	1.04906 (14)	0.37662 (11)	0.0350 (2)	
C14	−0.10538 (18)	1.23850 (15)	0.76265 (11)	0.0402 (3)	
C15	−0.1323 (2)	1.38051 (16)	0.68896 (13)	0.0507 (3)	
N16	−0.1573 (2)	1.49658 (16)	0.63094 (14)	0.0757 (4)	
C17	−0.2293 (2)	1.26880 (16)	0.89586 (12)	0.0459 (3)	
N18	−0.3258 (2)	1.29469 (17)	1.00271 (12)	0.0639 (4)	
C19	0.7624 (2)	0.67329 (19)	0.07853 (15)	0.0603 (4)	
H19A	0.8867	0.6900	0.0587	0.090*	
H19B	0.7228	0.6862	0.0026	0.090*	
H19C	0.7735	0.5623	0.1057	0.090*	

### Atomic displacement parameters (Å^2^)

**Table:**

	*U*^11^	*U*^22^	*U*^33^	*U*^12^	*U*^13^	*U*^23^
N1	0.0432 (5)	0.0282 (5)	0.0350 (5)	−0.0081 (4)	−0.0091 (4)	0.0063 (4)
C2	0.0378 (5)	0.0310 (5)	0.0351 (5)	−0.0105 (4)	−0.0126 (4)	0.0079 (4)
C3	0.0371 (6)	0.0354 (5)	0.0354 (6)	−0.0134 (4)	−0.0140 (4)	0.0074 (4)
C4	0.0483 (7)	0.0428 (6)	0.0390 (6)	−0.0122 (5)	−0.0131 (5)	0.0138 (5)
C5	0.0533 (7)	0.0390 (6)	0.0503 (7)	−0.0155 (5)	−0.0161 (6)	0.0180 (5)
C6	0.0508 (7)	0.0323 (5)	0.0477 (7)	−0.0084 (5)	−0.0161 (5)	0.0126 (5)
C7	0.0387 (6)	0.0325 (5)	0.0394 (6)	−0.0109 (4)	−0.0134 (4)	0.0084 (4)
C8	0.0372 (6)	0.0308 (5)	0.0394 (6)	−0.0102 (4)	−0.0119 (4)	0.0059 (4)
C9	0.0421 (6)	0.0322 (5)	0.0473 (7)	−0.0083 (5)	−0.0095 (5)	0.0034 (5)
C10	0.0411 (6)	0.0385 (6)	0.0435 (6)	−0.0111 (5)	−0.0075 (5)	−0.0010 (5)
C11	0.0436 (6)	0.0428 (6)	0.0383 (6)	−0.0153 (5)	−0.0081 (5)	0.0044 (5)
C12	0.0436 (6)	0.0345 (5)	0.0384 (6)	−0.0118 (5)	−0.0104 (5)	0.0087 (4)
C13	0.0364 (5)	0.0314 (5)	0.0355 (5)	−0.0104 (4)	−0.0110 (4)	0.0051 (4)
C14	0.0452 (6)	0.0370 (6)	0.0361 (6)	−0.0138 (5)	−0.0100 (5)	0.0040 (4)
C15	0.0575 (8)	0.0348 (6)	0.0448 (7)	−0.0081 (5)	−0.0038 (6)	0.0020 (5)
N16	0.0935 (10)	0.0380 (6)	0.0602 (8)	−0.0035 (6)	0.0026 (7)	0.0117 (5)
C17	0.0528 (7)	0.0391 (6)	0.0413 (6)	−0.0140 (5)	−0.0107 (5)	0.0027 (5)
N18	0.0785 (9)	0.0543 (7)	0.0450 (7)	−0.0193 (6)	−0.0046 (6)	0.0014 (5)
C19	0.0588 (9)	0.0472 (8)	0.0544 (8)	−0.0072 (6)	−0.0008 (7)	−0.0068 (6)

### Geometric parameters (Å, °)

**Table:**

N1—C13	1.3708 (15)	C8—C9	1.4088 (16)
N1—C2	1.3918 (14)	C8—C13	1.4106 (15)
N1—H1	0.86 (2)	C9—C10	1.3775 (18)
C2—C7	1.3906 (16)	C9—H9	0.9300
C2—C3	1.4265 (16)	C10—C11	1.4088 (17)
C3—C14	1.3747 (16)	C10—C19	1.5081 (18)
C3—C4	1.5040 (15)	C11—C12	1.3738 (17)
C4—C5	1.5209 (18)	C11—H11	0.9300
C4—H4A	0.9700	C12—C13	1.4004 (16)
C4—H4B	0.9700	C12—H12	0.9300
C5—C6	1.5150 (19)	C14—C15	1.4198 (17)
C5—H5A	0.9700	C14—C17	1.4340 (17)
C5—H5B	0.9700	C15—N16	1.1478 (18)
C6—C7	1.4884 (15)	C17—N18	1.1445 (17)
C6—H6A	0.9700	C19—H19A	0.9600
C6—H6B	0.9700	C19—H19B	0.9600
C7—C8	1.4172 (16)	C19—H19C	0.9600
			
C13—N1—C2	108.39 (9)	C9—C8—C13	119.72 (10)
C13—N1—H1	124.4 (13)	C9—C8—C7	133.50 (11)
C2—N1—H1	127.0 (13)	C13—C8—C7	106.76 (10)
C7—C2—N1	108.68 (10)	C10—C9—C8	119.53 (11)
C7—C2—C3	123.14 (10)	C10—C9—H9	120.2
N1—C2—C3	128.18 (10)	C8—C9—H9	120.2
C14—C3—C2	125.69 (10)	C9—C10—C11	119.15 (11)
C14—C3—C4	119.19 (10)	C9—C10—C19	121.77 (12)
C2—C3—C4	115.11 (10)	C11—C10—C19	119.08 (12)
C3—C4—C5	114.33 (10)	C12—C11—C10	123.24 (11)
C3—C4—H4A	108.7	C12—C11—H11	118.4
C5—C4—H4A	108.7	C10—C11—H11	118.4
C3—C4—H4B	108.7	C11—C12—C13	117.21 (11)
C5—C4—H4B	108.7	C11—C12—H12	121.4
H4A—C4—H4B	107.6	C13—C12—H12	121.4
C6—C5—C4	112.68 (11)	N1—C13—C12	130.09 (10)
C6—C5—H5A	109.1	N1—C13—C8	108.75 (10)
C4—C5—H5A	109.1	C12—C13—C8	121.14 (10)
C6—C5—H5B	109.1	C3—C14—C15	124.22 (11)
C4—C5—H5B	109.1	C3—C14—C17	120.91 (11)
H5A—C5—H5B	107.8	C15—C14—C17	114.87 (11)
C7—C6—C5	110.49 (10)	N16—C15—C14	178.80 (16)
C7—C6—H6A	109.6	N18—C17—C14	178.61 (15)
C5—C6—H6A	109.6	C10—C19—H19A	109.5
C7—C6—H6B	109.6	C10—C19—H19B	109.5
C5—C6—H6B	109.6	H19A—C19—H19B	109.5
H6A—C6—H6B	108.1	C10—C19—H19C	109.5
C2—C7—C8	107.42 (10)	H19A—C19—H19C	109.5
C2—C7—C6	123.45 (11)	H19B—C19—H19C	109.5
C8—C7—C6	129.13 (11)		
			
C13—N1—C2—C7	0.46 (13)	C8—C9—C10—C11	0.34 (19)
C13—N1—C2—C3	−179.37 (11)	C8—C9—C10—C19	−179.48 (12)
C7—C2—C3—C14	−176.13 (11)	C9—C10—C11—C12	0.3 (2)
N1—C2—C3—C14	3.69 (19)	C19—C10—C11—C12	−179.91 (13)
C7—C2—C3—C4	5.41 (16)	C10—C11—C12—C13	−0.68 (19)
N1—C2—C3—C4	−174.77 (10)	C2—N1—C13—C12	178.08 (11)
C14—C3—C4—C5	149.75 (12)	C2—N1—C13—C8	−0.20 (13)
C2—C3—C4—C5	−31.69 (15)	C11—C12—C13—N1	−177.58 (11)
C3—C4—C5—C6	52.95 (15)	C11—C12—C13—C8	0.51 (18)
C4—C5—C6—C7	−45.23 (15)	C9—C8—C13—N1	178.52 (10)
N1—C2—C7—C8	−0.54 (13)	C7—C8—C13—N1	−0.13 (13)
C3—C2—C7—C8	179.31 (10)	C9—C8—C13—C12	0.06 (17)
N1—C2—C7—C6	−179.76 (10)	C7—C8—C13—C12	−178.59 (10)
C3—C2—C7—C6	0.08 (18)	C2—C3—C14—C15	1.6 (2)
C5—C6—C7—C2	20.28 (17)	C4—C3—C14—C15	−179.95 (12)
C5—C6—C7—C8	−158.76 (12)	C2—C3—C14—C17	−178.17 (11)
C2—C7—C8—C9	−177.97 (13)	C4—C3—C14—C17	0.23 (17)
C6—C7—C8—C9	1.2 (2)	C3—C14—C15—N16	159 (8)
C2—C7—C8—C13	0.41 (13)	C17—C14—C15—N16	−22 (9)
C6—C7—C8—C13	179.58 (11)	C3—C14—C17—N18	105 (7)
C13—C8—C9—C10	−0.50 (18)	C15—C14—C17—N18	−74 (7)
C7—C8—C9—C10	177.73 (12)		

### Hydrogen-bond geometry (Å, °)

**Table:**

*D*—H···*A*	*D*—H	H···*A*	*D*···*A*	*D*—H···*A*
N1—H1···N16	0.86 (2)	2.59 (2)	3.3099 (17)	141.4 (16)
N1—H1···N16^i^	0.86 (2)	2.49 (2)	3.2150 (17)	142.8 (16)

Symmetry codes:  (i) −*x*, −*y*+3, −*z*+1.
